# Book Review

**Published:** 1910-12

**Authors:** 


**Memories of Eighty Years.** JOHN BEDDOE, M.D., LL.D., F.R.S. Pp. x, 322. Bristol: J. W. Arrowsmith. 1910.

Rarely does it happen that a worthy octogenarian autobiography is possible, but the writer of his own life history is the one person who can do it best, if his mental powers are equal to the task. Dr. Beddoe tells us, in his preface, that these memories have scarcely any basis of journal or record. It follows that his memory must be one of the best, and his mental powers unflagging.

This is all the more remarkable as he gives a graphic description of the difficulties of his friends in rearing him up to manhood. We read of him as " a sickly child," whose parents did not allow him to be taught to read or write, " often ailing in my childhood." Again, "My school days were much broken into by illness; an attack of rheumatic fever kept me long at home, one of typhoid followed, and later on dysentery." In spite of this he became, at seventeen, head of the school, Bridgnorth Grammar School, but " I had not strength for such a position." Games were abhorred, and finally " I broke down physically, and had to give up thoughts of the University." At twenty heart symptoms are mentioned. " I was forbidden to study," but in spite of this he read up Physiological Chemistry, in view of the dyspepsia from which he often suffered, and in anticipation of the modern method of calorics, he adopted a method of food valuation of his own. On the advice of Sir Charles Hastings he began to think that he would be better qualified to succeed in medicine than in law; accordingly was entered at University College, London, and this in spite of " the proverb that a physician does not get bread until he has no teeth wherewith to eat it. In my case it was literally fulfilled." After a winter of hard study we find mention of the onset of pulmonary symptoms; a year later he was still suffering from both pulmonary and cardiac troubles, and though active in habits, he was weak and the muscular development was poor. However, he learnt, at the age of twenty-four, that slow, deliberate climbing would strengthen an irritable heart, and we find him climbing the Cumberland mountains, sketching on Dungeon Fell and at the top of Harrison Stickle, 2,400 feet, also climbing a steep chimney on Scawfell Pike, and then triumphing over his comrades by running to see the Cathedral at Amiens during a halt of the train.

From this time onward we find little record of physical disability, but we note that at the age of fifty-nine he was spoken of as " our venerable friend." This description was very appropriate when he appeared in fancy dress as Melancthon or Avicenna, but the writer well remembers a remark of the late Dr. Marshall that " Beddoe had always been a Methuselah." Chapter IV contains the story of his life as one of the Civil

Medical Staff in Asia Minor and the Crimea. Chapter VII gives many anthropological reminiscences, and other chapters describe his travels in Ireland, Bretagne, Auvergne, and Australasia. The book is full of interesting stories, many of them very humorous and full of life. We can only offer our cordial congratulations to Dr. Beddoe that, in spite of his early years of weakness, he still retains a vitality far in excess of his years, and that he is able to give so good a story of his life conquest of infirmities and weakness from which many would have given up the contest. We trust he may maintain the contest for many years more, and give us further encouragement and instruction.

**Mentally Deficient Children.** G. E. SHUTTLEWORTH AND W. A. POTTS, M.D. Third Edition. Pp. xviii, 236. London: H. K. Lewis. 1910.—Dr. W. A. Potts, of Birmingham, has been called in to assist in writing the third edition of Dr. Shuttleworth's well-known manual, which has also been translated into French since the second edition appeared. A new chapter on the medical examination of children requiring special instruction has been added, and much new material has been incorporated in the other chapters. The report of the Royal Commission on Care and Control of the Feeble-minded has provided much of this, and the influence of the newly-introduced medical examination of school children is apparent throughout the book. The medical practitioner will find in this small volume a vast amount of information to help him in distinguishing between the various types of mental defect, and to guide him in the education of his patients; the social reformer will also find the broad economic aspects of the matter concisely treated. It is good to find how much can be done for the most deficient minds, and to see how thoroughly the public conscience is awakening to the inadequacy of the provision for suitable care and control in this country. The book is judiciously paragraphed, and easy to read. The authors have sinned in one small matter—the spelling of proper names: "Warren" Tay and "McGarrison," for example. In this respect, however, they are in distinguished company. Two excellent features call for special remark in conclusion: the book contains a number of good photographs and diagrams, and there is a bibliography in which all important contributions to the subject are included.

**Three Lectures on Epilepsy.** By W. ALDREN TURNER, M.D. Pp. x, 62. Edinburgh: James F. Mackenzie. 1910. **Epilepsia.** *Revue Internationale.* Official organ of the International League for dealing with Epilepsy. Published Quarterly. Parts I, II, III. London: Williams and Norgate. **Insanity in Every-day Practice.** By E. G. YOUNGER, M.D. Second Edition. Pp. viii, 124. London: Baillière, Tindall and

Cox. 1910.—Dr. Aldren Turner's Morisonian lectures are reproduced in booklet form, and constitute a lucid and concise account of the many problems in epilepsy, and a survey of the treatment of this malady. This small work will be found to contain much more information than its size suggests, and it should be very useful to the practitioner who wishes to be informed as to the latest views and results of research. *The Quarterly Review of Epilepsy* is devoted to a survey of international work done, and to the production of original contributions prepared for it. The large amount of literature in connection with epilepsy demands such a publication, which summarises the results of European and English research. The names of the editorial staff are guarantees that the articles both original and selected will be well chosen. Dr. Younger's book is a good brief work for the student and busy practitioner. It is clearly written, full of sound, practical instruction, and handy for reference. The author has been at pains to avoid controversial matter, and presents his types of insanity in the clear and discrete form essential to a short, practical work. The section on procedures under the Lunacy Acts should be of much service to the general practitioner.

**Index of Symptoms: with Diagnostic Methods.** By Ralph Winnington Leftwich, M.D. Fourth Edition. Bp. xiv, 451. London: Smith, Elder & Co.—This small volume demands more than a superficial examination. Its concise tabular form and general arrangement might lead a careless reader to consign it to the class of "cram" books. But as a matter of fact it is much more than this; it is a very successful attempt to classify symptoms under headings as a help to diagnosis, and the completeness and skill with which this has been done is worthy of great praise. Going carefully and critically through the pages it is difficult to find any errors either of omission or commission, and the short explanatory paragraphs are full of excellent matter, useful alike to the practitioner and the student. The brief but interesting account of some of the fallacies of diagnosis; the tables showing the incidence of sex, age, occupation and so forth in the causation of disease; the valuable "tips" as to the position of pain in the back, abdomen, etc., as indication of certain diseased conditions; the epitome of tests for urine, stomach contents, sensation, etc., etc., are all noticeable for their terseness, accuracy and clearness of style. It is a book that no amount of work in a library could render possible; it is obviously the outcome of extensive knowledge and experience, and as such may be considered as a guide, not merely a compilation. The author is fully justified in expressing the hope "that the indexing and collection of symptoms made for the first time in this book, will lead in the future to the erection of semeiology into something like a true science."

**Memories.** By a Hospital Nurse. Pp. viii, 168. Bristol: John Wright & Sons Ltd. 1910.—This little work, as stated in the preface, claims to be an account of a nurse's life in the wards and at private cases—simple narratives of very ordinary everyday occurrences, with occasionally thoughts and comments thereon, told in a plain and pious manner. Like other records of a somewhat similar nature, the story abounds in touches reminding one of Esther Summerson in *Bleak House*. Hidden, or only partially hidden, beneath the surface of humility is the implication of self-denial and goodness, a state of mind common enough amongst good women, but much disliked by most men, whether good or bad. The authoress, in fact, goes perilously near self-glorification. She appreciates clearly and well the domain of the nurse, but cannot resist the temptation of talking of matters that no nurse should feel confident to give an opinion on. She even goes so far as to say of lunacy patients: "All such sufferers with whom I personally have had to do recovered in a comparatively short time." There is an air of veracity about the trivial incidents narrated which gives an interest to the book, and probably many of the public, who like a glimpse into the medical or nursing worlds will appreciate and enjoy its perusal.

**The Rat and its Relation to the Public Health.** By Various Authors. Prepared by Direction of the Surgeon-General, Treasury Department, Public Health and Marine Hospital Service of the United States. Pp. 254. Washington: Government Printing Office. 1910.—This volume is opportune, and is by no means the least valuable of the many excellent publications issued by the department. The evidence which has been rapidly accumulating to prove that the rat and his parasites are responsible for the transmission of plague, and that plague itself is essentially a disease of the rat, calls for a complete survey of the life history of this genus, of the diseases peculiar to it, and of their position in regard to disease in man. All these points are dealt with in this volume by eighteen authors, each dealing with that aspect of the question of which he has made a special study. The result is a most interesting and authoritative presentment of an important subject in public health, and it is well to note that the practical aspects of prevention have not been overlooked. The difficulties of rat destruction, the comparative inefficacy of bacterial viruses in exterminating rats, the primary importance of effective "rat-proofing" of houses, and the shipping aspects of the rat question, receive consideration in turn. The volume is well worth careful study.

